# Challenging Reaction Prediction Models to Generalize to Novel Chemistry

**DOI:** 10.1021/acscentsci.5c00055

**Published:** 2025-03-12

**Authors:** John Bradshaw, Anji Zhang, Babak Mahjour, David E. Graff, Marwin H. S. Segler, Connor W. Coley

**Affiliations:** † Department of Chemical Engineering, Massachusetts Institute of Technology, Cambridge, Massachusetts 02139, United States; ‡ Department of Chemistry and Chemical Biology, Harvard University, Cambridge, Massachusetts 02138, United States; § Microsoft Research AI for Science, Cambridge CB1 2FB, United Kingdom; ∥ Department of Electrical Engineering and Computer Science, Massachusetts Institute of Technology, Cambridge, Massachusetts 02139, United States; ¶ Department of Chemistry, Massachusetts Institute of Technology, Cambridge, Massachusetts 02139, United States

## Abstract

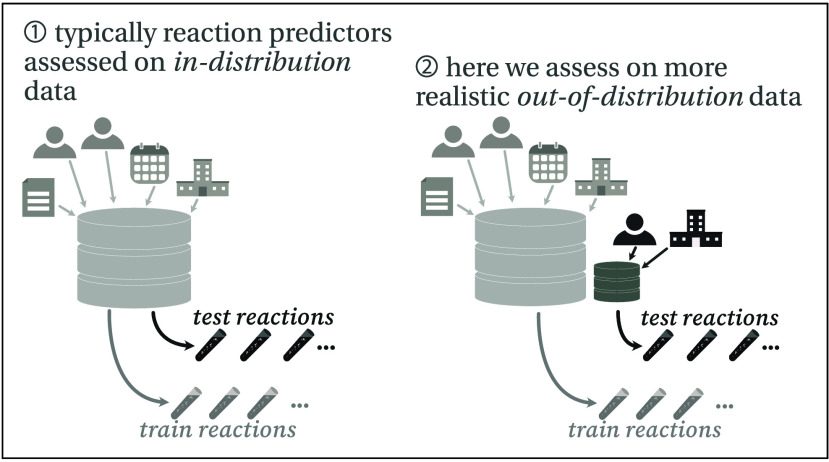

Deep learning models for anticipating the products of organic reactions have found many use cases, including validating retrosynthetic pathways and constraining synthesis-based molecular design tools. Despite compelling performance on popular benchmark tasks, strange and erroneous predictions sometimes ensue when using these models in practice. The core issue is that common benchmarks test models in an *in-distribution* setting, whereas many real-world uses for these models are in *out-of-distribution* settings and require a greater degree of extrapolation. To better understand how current reaction predictors work in out-of-distribution domains, we report a series of more challenging evaluations of a prototypical SMILES-based deep learning model. First, we illustrate how performance on randomly sampled data sets is overly optimistic compared to performance when generalizing to new patents or new authors. Second, we conduct time splits that evaluate how models perform when tested on reactions published years after those in their training set, mimicking real-world deployment. Finally, we consider extrapolation across reaction classes to reflect what would be required for the discovery of novel reaction types. This panel of tasks can reveal the capabilities and limitations of today’s reaction predictors, acting as a crucial first step in the development of tomorrow’s next-generation models capable of reaction discovery.

## Abstract 2

Despite excellent benchmark performance, ML models for reaction prediction can struggle on real-world data—we evaluate these limitations by challenging a model on different out-of-distribution tasks.

## Introduction

**Figure 1:**
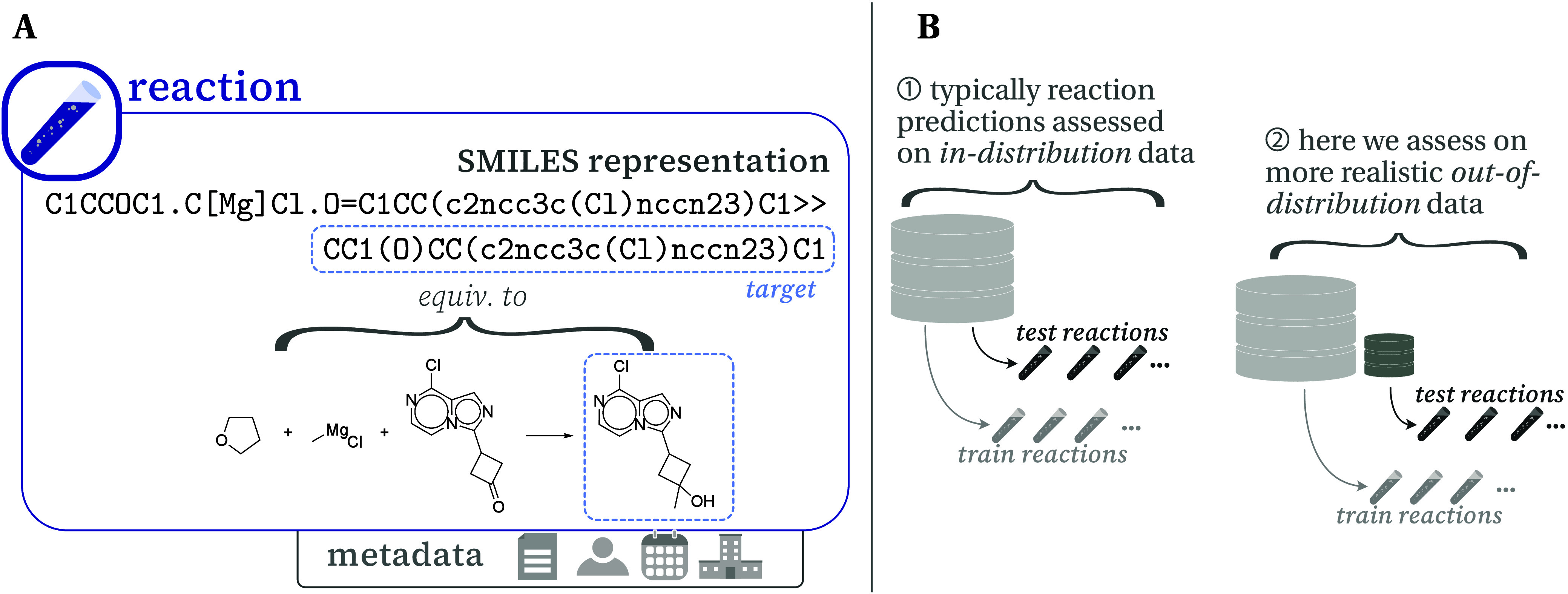
(A) Reaction prediction, in the context of this manuscript, is the task of predicting the major product(s) of a reaction given the reactants. (Note that by “reaction” we mean specific reported reaction examples, rather than generic reaction “types” or “classes” that cover a large group of related specific examples—we will come back to the concept of reaction types in a later section.) Chemical reaction data sets are often curated from academic or patent literature, so each reaction is associated with a set of hidden metadata (the predictive model does not see this), such as the reaction’s associated patent document, its authors, its publication date, the assignee/organization that filed the patent, etc. (B) Typically, reaction predictors are assessed in an in-distribution setting, meaning that the training and test reactions come from the same distributions. However, in the real world, reaction prediction models are often deployed on out-of-distribution data, a setup that we will discuss how to replicate.

Reaction prediction—the task of anticipating *in silico* the products of a chemical reaction given the reactants (Figure 1A; refs (1−4))—is a crucial technology in (a) the validation of retrosynthetic pathways,^5−8^ (b) as a component of synthesis-based de novo design algorithms,^9−18^ and potentially (c) for the discovery of new reactions.^19−23^ Encouragingly, there has been a burst of recent works developing a variety of machine learning–based reaction predictors that achieve very high accuracies on common benchmark tasks.^3,24−36^ With the best of these models matching or outperforming human chemists (see, e.g., ref (25), §4.2) and reporting top-5 accuracies above 95% (meaning that the correct answer is found in the top five predictions of the model over 95% of the time; see, e.g., ref (34), p.9), performance seems to have saturated. Distinguishing best-performing models has become challenging. It is also natural to wonder if the task of reaction prediction has been “solved” to a meaningful degree.

When using these models in practice, it quickly becomes apparent that the answer is a resounding no. In fact, when using reaction predictors in new domains, not only might a model make an incorrect prediction, it might hallucinate a product preposterous to a human chemist. The discrepancy between the reported performance on benchmarks with the subjective performance that can be seen in practice can be explained by the setting in which the model is evaluated. Benchmark tasks (such as USPTO_Stereo, USPTO_MIT, Pistachio, etc.^25,37−39^) evaluate models on in-distribution (ID) data, where the reactions in the test set come from the same distribution as that used to train the model, for example, using a random partition of a reaction data set. However, in practice we often want to evaluate a model on out-of-distribution (OOD) data, meaning the test reactions are sampled from a different distribution than that used to train the model (Figure 1B). In fact, using these models for reaction discovery is by definition an out-of-distribution task.

The unrealistic nature of current evaluations not only robs us of a sense of how existing methods perform, but it does so in such a way that overstates performance, stymieing analysis of where methods fall short and how to improve them. To address this, we reassess what it means to evaluate a reaction predictor. We discuss and develop new tasks to test how well reaction predictors can do in different out-of-distribution domains, investigating when and how they are able to generalize and extrapolate in such settings. Concretely, we seek to answer the following questions:

1. How overoptimistic are the random splits that are currently the most popular style of split for this task, and what is a more realistic evaluation of a reaction predictor’s performance?
2. If we want to use reaction predictors trained today on future data sets, how should we design benchmarks to test models prospectively?
3. When, and under what circumstances, might reaction predictors be able to discover new reactions?

## Results

### Random Splits Are Overoptimistic by Ignoring Data Set Structure

Typically, reaction predictors are tested on *random splits*. That is, we treat a large reaction data set (usually extracted from the patent literature^37,39,40^) as independently and identically distributed and randomly divide the reactions up between the training, validation, and test sets. The reactions in the training and validation sets are used for deciding on hyperparameters and for training the model (in what follows we will make no further distinction between the training and validation sets), while those in the test set are used for the final evaluation.

**Figure 2:**
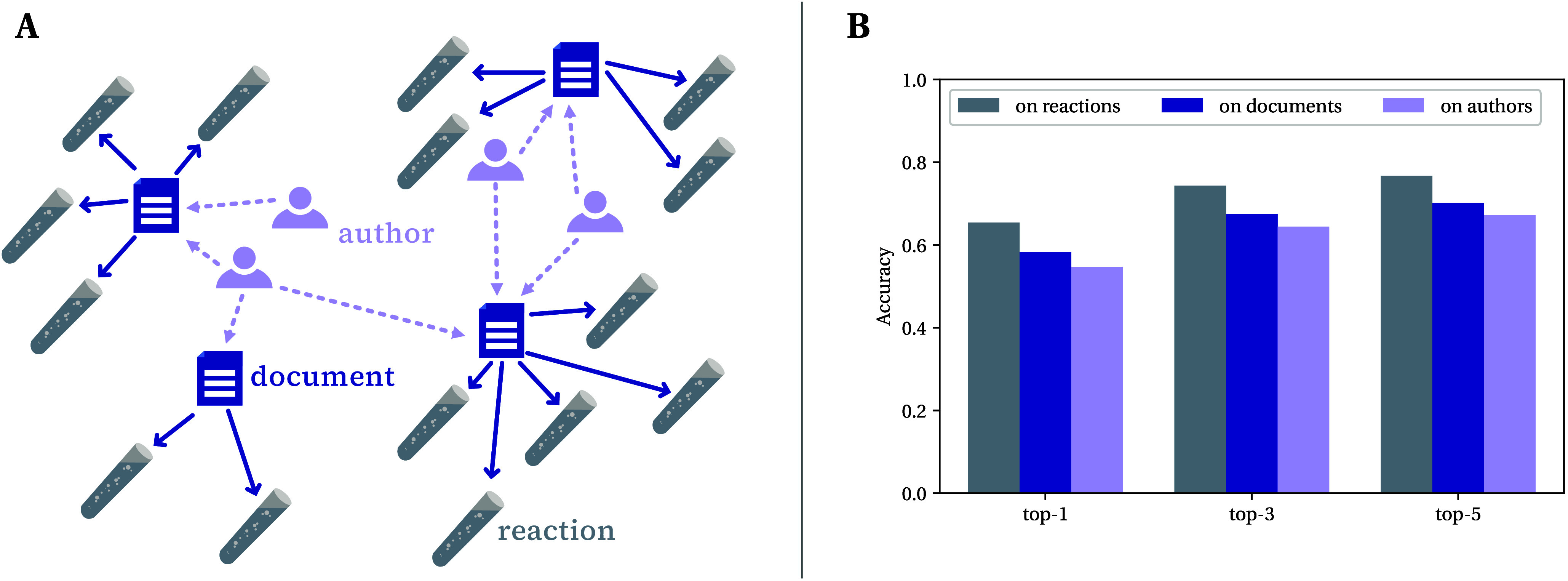
(A) Reaction data sets are formed by authors coming together and writing documents, which contain many (often similar) reactions. Evaluating a reaction predictor on train/test sets that account for this structure provides different accuracy scores. (Note that in this paper we clean and deduplicate reactions before creating the splits, such that a reaction is only associated with one document—see the Supporting Information.) (B) Top-1, 3, and 5 accuracies when doing reaction- (i.e., random), document-, and author-based splits.

However, the universe does not actually generate the reactions in reaction data sets independently! Instead it generates chemists, who join organizations, form teams, and write documents (e.g., journal articles, patents, etc.) containing reactions (Figure 2A). Often these documents contain many related reactions, for instance, a group of different reactants undergoing the same transform in an exploration of reaction scope or structure–activity relationships (SAR). By creating a random split, many of these highly related reactions will be spread across the train and test sets. This in turn means that when making test predictions, the model can take advantage of the highly similar reactions that ended up in its training set, a scenario less likely to occur if one were truly independently sampling reactions for testing.

In order to better understand how this data set structure affects the accuracy metrics of reaction predictors, we investigate alternative splits of the Pistachio data set^39,40^ that take this structure into account (while we cannot share the created splits used due to the proprietary nature of the Pistachio data set, the code for recreating them is available at https://github.com/john-bradshaw/rxn-splits). In particular, we compare three different strategies for dividing reactions into the training and test splits: (1) *on reactions*, which is the same as a typical random splitting strategy; (2) *on documents*, which means that all reactions associated with each document end up together either in the training or test set; and (3) *on authors*, which is similar to the document-based approach but done on authors instead, such that each author (and their corresponding reactions) is associated with either the training or test set.

We train separate language-based reaction predictor models on each of these splits, controlling for data set size, and present the accuracy results in Figure 2 (and Table S2 in the Supporting Information). Specifically, we use an encoder-decoder Transformer model,^41^ based on the BART architecture,^42^ using the SMILES-based tokenization scheme proposed by Schwaller et al.^38^ In practice, this BART model is very similar to the Molecular Transformer model,^3^ with a few small architectural differences. We train the model using the AdamW optimizer^43^ and use beam search (with a beam width of 5) at inference time. Full details of the model and the training setup can be found in the Methods section of the Supporting Information (and code can be found at https://github.com/john-bradshaw/rxn-lm). Evaluation focuses on the top-*k* accuracy metric, which asks whether the experimentally recorded major product appears in the *k* highest ranked predictions by the model (this comparison is done on SMILES after canonicalization).

Figure 2 confirms that traditional, random splits (*on reactions*) are overoptimistic relative to splitting on documents or authors. We see that a model trained and evaluated on an *on reactions* split obtains a top-1 accuracy of 65%. When instead splitting on documents, the same model obtains a lower accuracy of 58%, which further drops to 55% when splitting on author. Similar trends are also found when looking at the top-3 and top-5 accuracies. Overall, this indicates that the group of similar reactions associated with the same document or author leads to better reported model performance when these reactions are spread across both the training and test sets. When using a reaction predictor “in the wild”, one is unlikely to be evaluating on reactions that are in a document already used to train the model, and so these document- and author-based splits are more likely to represent real-world performance. The drop of ∼10% accuracy (on author-based splits) is therefore important not only in giving a better sense of current ML-based reaction predictor performance, but also in highlighting that there is still more room left for improvement than suggested by previous benchmarks.

### Time-Based Splits Enable Prospective Evaluation Mimicking Real-World Use

The document- and author-based splits considered in the previous section help provide a stricter evaluation of reaction predictors when they are used *retrospectively*, i.e., to make predictions for inputs similar to already discovered and documented reactions. This can be the case, for instance, when checking the credibility of reactions in proposed synthesis plans, as the reactions will often be close (i.e., having similar reactants or similar transforms) to those already known, particularly if one wants confidence in their practicality. However, often it is also important to know how well reaction predictors work *prospectively*, i.e., on reactions of interest going forward, which might involve a different distribution of reaction types or substrates. For example, when assessing the utility of reaction predictors for reaction discovery, one only wants to know how well they would work on new, undiscovered transforms.

**Figure 3:**
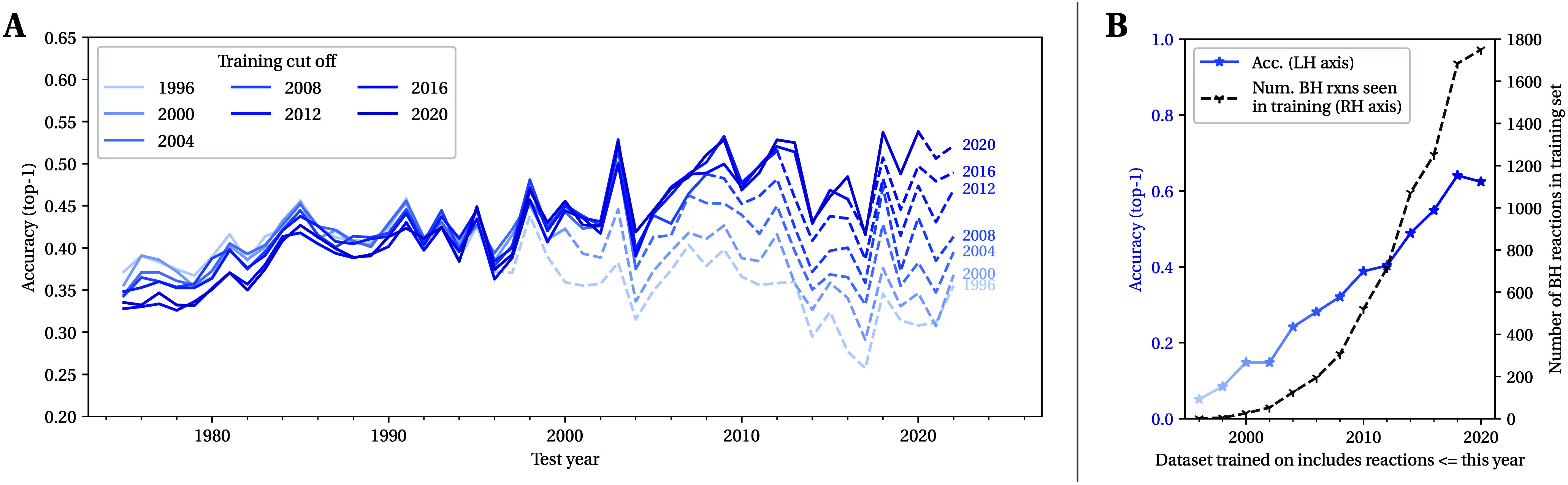
(A) Top-1 accuracies of reaction predictors trained up to different time cutoffs (different colors) when evaluated on held-out test sets for each year (*x*-axis). For instance, the line in the lightest shade, marked “1996”, reports the top-1 accuracy for a reaction predictor trained on reactions that were reported up to 1996 (inclusive). The dashed line indicates model performance when the model is “extrapolating”—meaning that the test set year is beyond the model’s time cutoff. Note that we control for training set size so each model sees the same number of reactions in training (the absolute performance of the model is therefore lower than when training on all available data up to a given year). Further details on experimental setup and additional results can be found in the Supporting Information. (B) Performance of the models trained on different time splits on a separate, static test set of Buchwald–Hartwig reactions. The blue solid line shows the top-1 accuracies (left-hand axis), while the dotted gray line shows the number of Buchwald–Hartwig reactions in the models’ training sets (right-hand axis).

To evaluate how well reaction predictors work prospectively, we create a *time-based* split. Rather than just considering a single time-based split, as is typical,^44^ we instead create a series of splits to consider how model accuracy changes as the difference in time between the training and test set increases. Specifically, this process works as follows: First of all, we take the same processed Pistachio data set used in the previous section, which contains reactions from patents as early as the 1970s, and split off a separate held-out test set for each year. Next, we take the remaining reaction data and create a sequence of training sets with different time cutoffs, each only containing reactions recorded up to and including in the associated cutoff year. Finally, we evaluate models trained on these different training sets on our held-out test sets (Figure 3A). When creating these training sets, we control for training set size (results without this restriction are shown in Figures S5 and S6).

Figure 3A shows several important trends. Looking first at the performance of a single model in the interpolative regime, we see that while there is some interyear variation—in part due to variability from the random selection of test sets—performance gradually increases until the model reaches its training cutoff point. We hypothesize that this effect is due to the shifts in the distributions of reaction types reported over time—the reaction types present in later years are more popular (see Figure S1 or refs (45 and 46)) and so better predicted.

Secondly, the interpolative regime reveals that the models trained on earlier cutoffs perform better on the earlier years (particularly from 1975–1990). This effect is linked with us controlling for the training set size: The models with the earlier cutoffs will have better specialized on reaction types present in the earlier time period because their fixed data budget is spread across a smaller range of years. When we remove this control (see Figure S5 in the Supporting Information) and train each model on all of the data available up to its cutoff, this second trend disappears.

Switching to analyzing the extrapolative regime (indicated by the dashed line), we notice that performance starts falling after the model’s training cutoff. Moreover, the drop in performance seems to be correlated with the difference between the training cutoff and test year, such that the larger the difference, the worse the performance. As such, we can think of the time axis as acting as a proxy for an “extrapolation distance”. Despite this drop in accuracy, the model trained on reactions reported up to 1996 still correctly predicts some reactions reported nearly 25 years later. This is likely because there are plenty of reactions (such as the Suzuki coupling) that are popular and remain popular; overall, this suggests that current methods have some utility in predicting the products of reactions conducted in the future.

#### Reaction Discovery and Adoption in a Time-Based Split

One factor that the extrapolation distance represents in a time-based split is the discovery and then widespread adoption of a new reaction; this is part of the reason why extrapolating into the future may be hard. We can assess this further by looking at the performance of the models trained on the time-based splits on specific reactions discovered during the time horizon we consider, for instance the Buchwald–Hartwig reaction, which was first reported in 1994.^47−49^ To do this, we define a new test set of all the Buchwald–Hartwig reactions that are not present in any of the models’ training sets (see the Supporting Information for more details), and then evaluate the models trained on each of the cutoffs on this new test set (Figure 3B and Table S3). Note that, in Figure 3B, the *x*-axis now represents the training set cutoff year (which was previously represented by the different colored lines in Figure 3A).

From this analysis, we see that the earliest model (trained on reactions that were first reported up to 1996) starts with only one Buchwald–Hartwig reaction in its training set. (While the Buchwald–Hartwig reaction was first published in the academic literature in 1994, our models are trained on data extracted from the patent literature, and so even by 1996 very few Buchwald–Hartwig reactions had been used.) The model obtains a low top-1 accuracy of 5.2% (8.2% top-5) on the held-out Buchwald–Hartwig reaction test set, reflecting the lack of knowledge the model has on this reaction class at this point in time. When we look at the models trained on later cutoffs, we see that they have seen far more Buchwald–Hartwig reactions in their training sets, and this corresponds to far higher accuracies (i.e., top-1 accuracies above 60%). Therefore, as expected, the ability to predict particular types of reactions depends on the availability of training data covering those types. The demonstrated difficulty of model extrapolation to unseen transforms can partly explain why accuracy falls off as the extrapolation distance increases (Figure 3A). Nevertheless, while the model accuracy starts off low, it is not zero, interestingly suggesting that some knowledge of this transform can also be inferred from the other reactions present.

### Reaction-Type-Based Splits Evaluate the Strictest Form of Extrapolation across Classes

If time-based splits serve partly as a surrogate for assessing performance on unseen reaction types, why not just evaluate on this task directly? In fact, reaction discovery is likely not the only factor that occurs in a time-based split, as there are also shifts in the conditions used and the chemical space being explored (we come back to investigating the differences between our splits later). Here, we instead assess the potential for successful generalization to new reaction types by using *NameRxn splits*.

NameRxn is a rules-based, hierarchical method of classifying reactions.^50,51^ The hierarchy is inspired by and linked to other classification schemes^52,53^ and has been used for analyzing chemical trends.^45^ The NameRxn hierarchy consists of three levels, the first dealing with high-level categories (e.g., classes include “*3. C–C bond formation*”, “*4. heterocycle formation*”, etc.), with the subsequent two levels then further filtering this down. To illustrate, below “*3. C–C bond formation*” the second level contains categories such as “*3.1 Suzuki reactions*”, “*3.2 Heck reactions*”, etc., and the third level below “*3.1 Suzuki reactions*” contains the final classes “*3.1.1 Bromo Suzuki coupling*”, “*3.1.2 Chloro Suzuki coupling*”, etc. The three digit code associated with each NameRxn in the bottom level (e.g., “3.1.2” for “*Chloro Suzuki coupling*”) indicates exactly where in the hierarchy the NameRxn class lies.

Our reaction-type splits involve holding-out one or more of these NameRxn classes from the training set of our model to use as a test set. By doing this experiment for different NameRxn classes, we can get an idea of the “chemistry” the model can learn from other classes present in its training set and what forms of extrapolation it struggles with. Here we evaluate on five different reaction-type splits, holding out respectively, all (1) Grignard Ester, (2) Heck, (3) Chloro Suzuki, (4) Triflyloxy Suzuki, and (5) All Suzuki coupling reactions. Note that we consider three different classes of Suzuki splits: the Chloro Suzuki and Triflyloxy Suzuki splits consider subtypes of Suzuki reactions containing specific functional groups, whereas the All Suzuki split holds out all types of Suzuki reactions. Further details on the exact NameRxn classes each split entails is provided in the Supporting Information.

**Figure 4:**
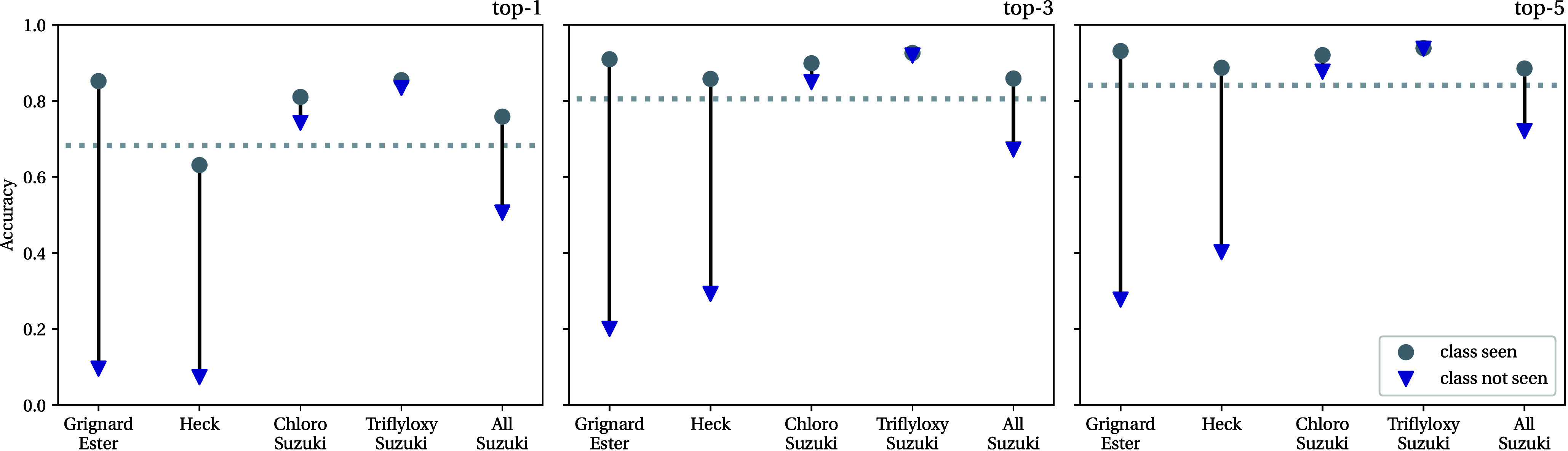
Top-1, 3, and 5 accuracies for reaction predictors evaluated on different reaction-type splits. Each column shows the accuracy on a held-out set of a particular reaction class both (a) when seeing 1000 separate examples of the same reaction type during training (gray circles, ●; intrinsic difficulty) and (b) when seeing no reactions of that type during training (blue triangles, ▼; extrapolation difficulty). The gray dashed horizontal line shows the accuracy of a reaction predictor evaluated on an in-distribution test set (i.e., containing many different reaction classes). Note that we remove all uncategorized reactions (NameRxn class “0.0”) when creating our data sets.

It is important to note that some reaction types can be *intrinsically hard* to predict even when the model has seen other example reactions from the same class due to the innate complexity of that transformation (for instance, a given reaction may yield multiple possible products or stereoisomeric products). However, our focus is on how hard the reaction types are to *extrapolate to*, which is a distinct concept. To assess this for each NameRxn split, we divide the group of held-out reactions in two: we add the first subset (1000 reactions) to the training set for a baseline model, while reserving the remainder for testing only. The baseline model’s performance on the test set can give us an idea of the reaction type’s intrinsic difficulty, and so puts the extrapolation performance in context (Figure 4 and Table S4).

Overall we see some small variation in the classes’ intrinsic difficulties, with the predictions on the Heck reactions in particular being marginally less accurate than those for the other classes. When it comes to extrapolation difficulty, the Suzuki splits, particularly the Chloro Suzuki and Triflyloxy Suzuki splits, appear the easiest. In contrast, the Grignard Ester and Heck reactions prove more challenging when reactions of those types are fully omitted from training. Interestingly, even with these harder splits, the models still demonstrate some ability to extrapolate, particularly when we look at the top-3 and top-5 predictions.

### Deeper Investigation into What Enables Reaction Class Extrapolation

**Figure 5:**
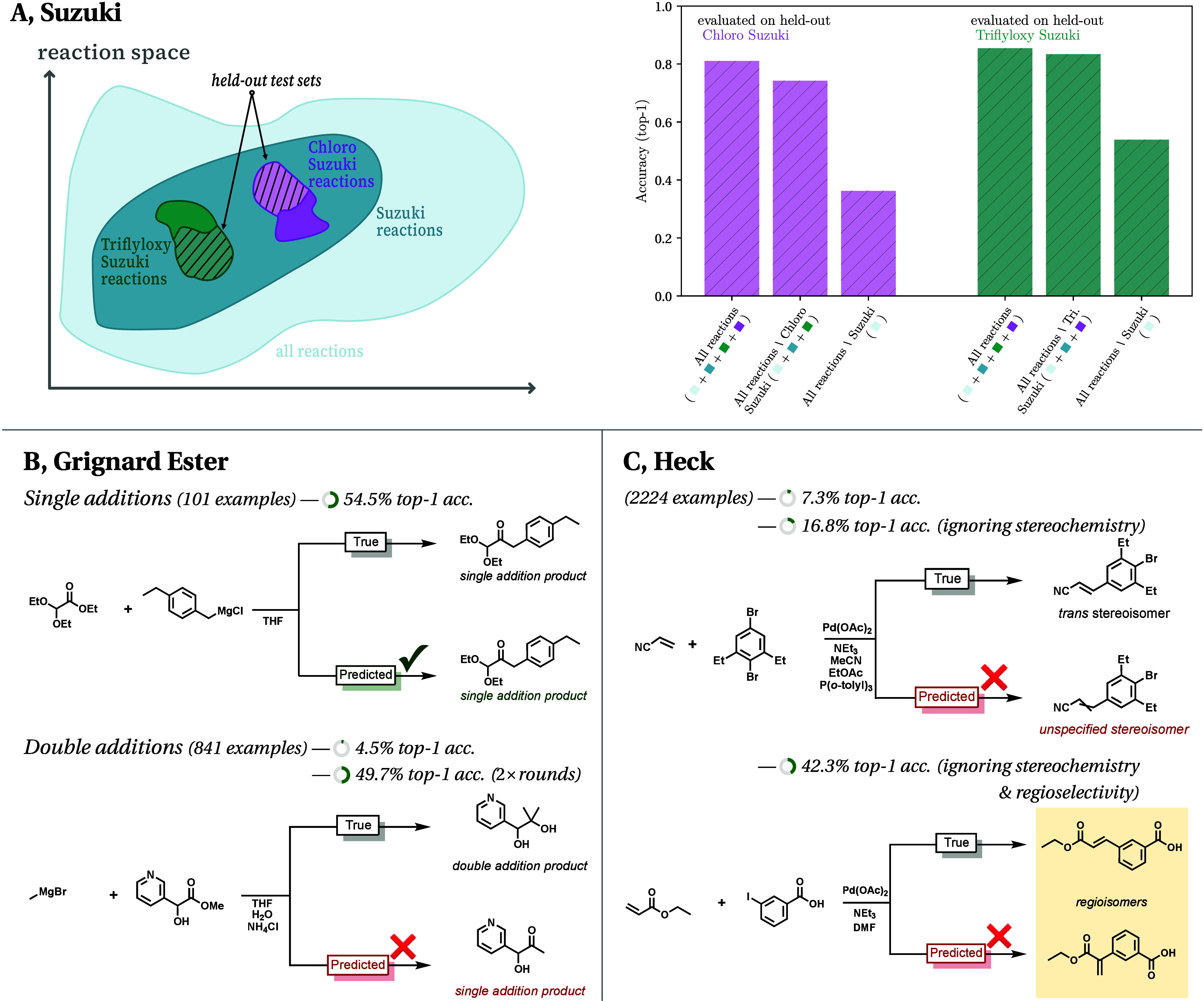
We investigate reasons for the contrasting performance in the different NameRxn splits. (A) For the Chloro Suzuki and Triflyloxy Suzuki splits, we assess whether the large number of other Suzuki reactions present can explain the good extrapolative performance. Namely, to evaluate on our specific Chloro and Triflyloxy Suzuki test sets, we create three different training sets (as shown by cartoon, left): (i) reactions from all classes (including separate reactions from the same specific Suzuki class); (ii) reactions only from other specific Suzuki reaction classes (and non-Suzuki reactions); and (iii) non-Suzuki reactions only. Results are shown on the right. The different bars show the accuracy for the different cases (the square color boxes in the *x*-axis labels indicate the reaction classes used in training the respective models). (B) For the Grignard Ester split we notice that the model does particularly poorly on a *double addition* subset, but such a reaction can actually be expressed as two single additions and that when we allow our model to do two rounds of predictions (i.e., when we feed the predicted product from the first round in as an input the second time around) performance improves. (C) For the Heck split we see that the model particularly struggles with the stereochemistry and regioselectivity present in these reactions (see text for further details).

We can further investigate how the model is able to extrapolate in these splits, and conversely why sometimes it is unable to do so. We look first at the Chloro and Triflyloxy Suzuki splits. Remarkably the model finds it easy to extrapolate to these classes: the drop in top-1 accuracy is less than 10%. We hypothesize that this is in part due to the large number of other Suzuki reactions that remain in the model’s training set, and so we investigate how excluding these reactions (using our All Suzuki split) affects results (see Figure 5A). Figure 5A confirms that removing these other Suzuki reactions has a far more pronounced effect on the extrapolation difficulty than removing reactions in the specific subclass, suggesting that the model does indeed learn from the Suzuki class as a whole. Having said that, even in this more challenging extrapolation, accuracy does not drop to zero, which could be attributed to the existence of many structurally similar non-Suzuki cross-coupling reactions, such as the Kumada coupling, Negishi coupling, Stille coupling, and others.

Second, analyzing the Grignard Ester reactions, we find that they can be divided into two main groups: single and double additions (see Figure 5B). Our model particularly struggles to predict the latter of these, exhibiting a top-1 accuracy of 4.5% on the double additions compared to the baseline model’s 91.1% (on the single additions both the model and the baseline achieve around 55%). This can be explained because most of the data set’s remaining (i.e., non-ester) Grignard reactions form single-addition products via the protonation of a stable tetrahedral magnesium alkoxide intermediate. In the Grignard Ester examples, however, this species collapses to give a carbonyl group that is typically more reactive than the ester starting material and thus in most cases reacts again with the Grignard nucleophile, yielding the double addition product. Thus, double additions can be mechanistically described as the continuation and repetition of a single addition reaction, and one therefore could model them as the composition of two single addition reactions. We reframed this task accordingly by feeding the product from the initial prediction back into the model as a reactant. With this change, the model improves from 4.5% to 49.7% top-1 accuracy on the double additions, showing that the latent chemical knowledge needed to predict Grignard Ester products already exists in the model, but the knowledge of what should be considered a *single* reaction step is not.

In the case of the Heck reaction split, many incorrect predictions are a result of the multiple possible isomeric products this reaction can produce: *E-* or *Z-*stereochemistry occurs due to the double bond formed, while different regioisomeric products can also result depending on factors such as the type of catalyst. To quantify how well the model deals with such nuances, we first re-evaluate the top-1 accuracy after removing all stereochemical information from both the predicted and ground-truth products. When doing so, we find that both the Heck-withheld and baseline models’ accuracies improve (from 7% to 17% and 63% to 85%, respectively), suggesting that stereochemistry may be intrinsically harder to predict rather than merely challenging to extrapolate to (which could clarify the baseline model’s relative performance in Figure 4).

Extending this analysis to regiochemistry, the top-1 accuracy of the Heck-withheld model further increases to 42% when different regioisomers are counted as correct predictions (Figure 5C), while the baseline model’s accuracy increases to 89%. Overall this confirms that the model’s performance on the Heck reaction can in part be attributed to the wide range of possible Heck products, and that this challenge is significantly amplified in extrapolative settings, where trends in regioselectivity in particular are hard to model without seeing the reaction class.

### Analysis of Distribution Shifts Offers Insights into the Relationship between Split Types

In the analysis done so far, we have taken an *objective-driven* approach to discussing and evaluating reaction extrapolation. That is, if your objective is to better understand how reaction predictors work on already explored reaction spaces, then use a document- or author-based split; if your objective is to understand how reaction predictors work prospectively, then use a time-based split; or if your objective is to understand how well reaction predictors can generalize to new types of transforms, then use a reaction-type split.

However, this is not the only approach one can take when designing out-of-distribution tasks. An alternative would be to take what we would call a *data-driven* approach to split design (we discuss relevant related work below and also in the next section). In particular, this looks at the data given to the model—typically focusing on the inputs—and picks a particular feature of this data to split on that ideally is not important for prediction; in certain cases it might even generate synthetic out-of-distribution data by editing existing data in a way that should not affect the label. Examples of data-driven approaches include adding Gaussian blur to images on classification tasks,^54^ splitting on molecular structure in molecular classification tasks,^55^ or splitting by molecular weight in reaction prediction tasks.^56^ Taken to the extreme, adversarial examples^57−60^ are out-of-distribution data specifically designed from the input data to be as challenging as possible to the model. Although the resultant splits from a data-driven approach can end up testing similar properties of the model, the approach differs in how the splits are derived.

**Figure 6:**
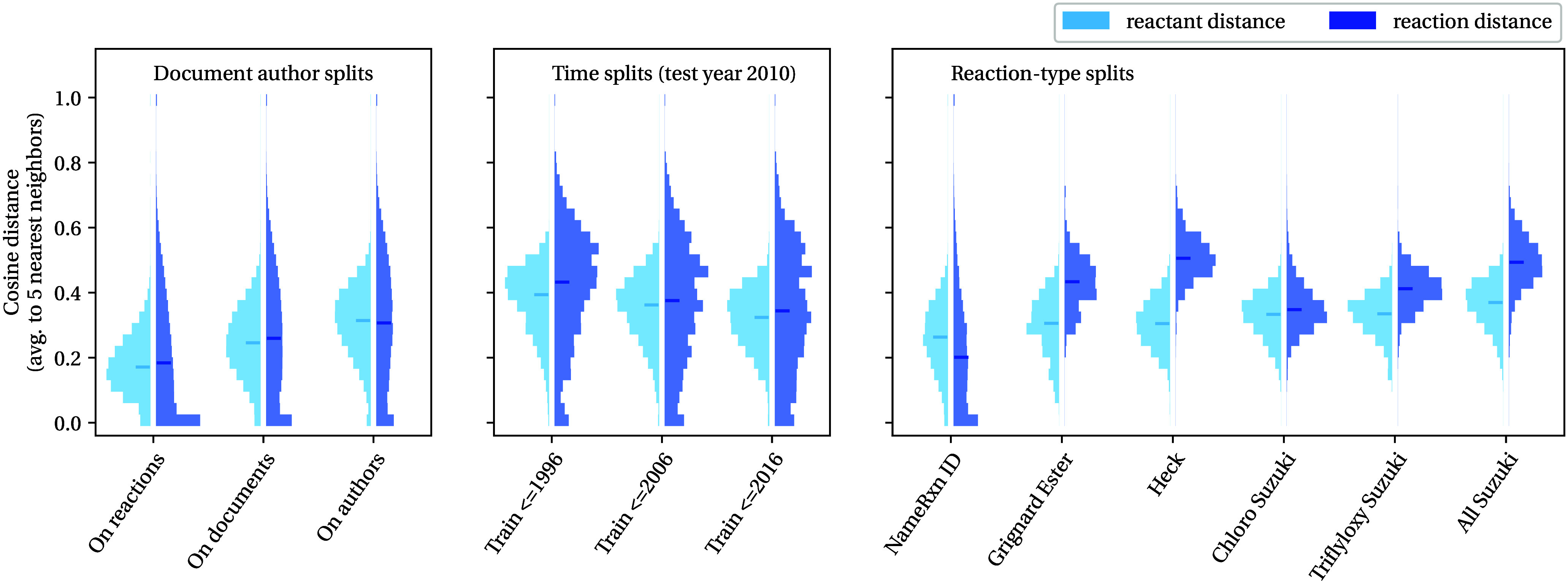
Distribution of the average cosine distance between each reaction in each test set to its nearest five neighbors in the corresponding training set. The left-hand part of each histogram (in the lighter blue) shows the distance in reactant fingerprint space (i.e., it indicates how different the test reactant molecules are to those the model sees in training), whereas the right-hand part of the histogram (in the darker blue) shows the distance in reaction fingerprint space (i.e., it indicates how different the reaction transform is from those seen in training). The solid horizontal lines indicate the median of each distribution. Further details about splits and how fingerprints are calculated can be found in the Supporting Information.

It is worth reassessing the splits we have considered from this point of view and comparing them in terms of how the input data differ between the training and test sets. When looking at the input to a reaction predictor, one can change it in two main ways. One can either consider (a) varying the reactants, i.e., consider performing the same reaction over different areas of reactant space, or (b) varying the transform, i.e., consider performing very different reactions over similar areas of reactant space. In Figure 6 we visualize how the splits vary along these two dimensions by plotting the distribution of distances from each test point to its five nearest neighbors in the training set in terms of (a) reactant fingerprint distances and (b) reaction fingerprint distances. While structural fingerprints do not provide a complete picture of how reactions may differ along these different dimensions, they are still useful as a quick-to-compute and practical guide.

Figure 6 shows that the distance to the nearest neighbors increases in both reactant and reaction fingerprint space on our out-of-distribution data sets, as one might expect. For the document- and author-based splits, we see a large shift in the reactant distance distribution (particularly when looking at the medians) and also a drop in the number of neighbors at zero distance in reaction space. Many patents contain multiple examples of one reaction type applied to many structurally similar reactants. The changes seen in distributions match what we might expect as we prevent the model from training and testing within these same substrate scopes, to training and then testing on different ones, highlighting what makes these splits more challenging.

The distributions for the time-based splits demonstrate that both the reactants and reactions change together as the model extrapolates further into the future. This differs from the reaction-type splits, where the distance in reaction space is greater than in reactant space, reflecting the design of these splits. Interestingly, the distance also seems somewhat correlated with the observed empirical difficulty of the split, with further distances seen more often for the harder Grignard Ester, Heck, and All Suzuki splits compared to the easier Chloro and Triflyloxy Suzuki splits. Therefore, such distances could be used to design further challenging splits.

## Discussion

### OOD Benchmarks Enable OOD Improvement

The importance of considering the data generating process and evaluating on OOD data has long been prevalent in ML more generally (see e.g., Figure 5 of ref (61) or refs (62−65)), with related developments including both better benchmarks^55,66−69^ and modeling/algorithm advancements to better deal with such data.^54,70−72^ Within the domain of ML for chemistry, OOD splits—such as scaffold, time, and others—have facilitated the development of more robust models for molecular regression and classification tasks.^44,73−77^

### OOD Benchmarks in Reaction Prediction

When it comes to reaction prediction, many models are introduced then tested only on in-distribution splits. Performance is sometimes broken down into how well methods do on subclasses of reactions (see, e.g., Table 5 of ref (3) or Figure 6 of ref (34)), but this is carried out in situations where the models have seen the same classes in their training set (i.e., an in-distribution setting). Sometimes single time-based,^78^ template,^79^ or document^28^ splits have been used, but this practice is not widespread. Alternative metrics to top-1 accuracy to evaluate methods have been studied in the context of in-distribution settings^8^ as well as works developing and discussing how to better deal with noisy reaction data.^80−82^

When OOD tasks have been investigated in reaction prediction, this is often in the context of method development for enabling models to adapt to new reaction types or different, often proprietary data sets.^22,83−86^ Techniques range from multitask learning to fine-tuning, but on the whole, evaluation is carried out on only a single type of split, often representing single reaction classes (for instance, Wang et al.^85^ focuses on the Heck reaction and Su et al.^84^ looks at the Chan–Lam coupling and how this relates to other, similar reaction classes).

Perhaps closer to the ideas here, there has also been a series of recent work reappraising how well existing reaction predictors models work: both in the forward^56,87,88^ (like our work here) and the inverse direction.^89,90^ For instance, Gil et al.^56^ proposed a new open-source benchmark for reaction prediction that includes a variety of new metrics, such as OOD accuracy on molecular weight–based splits as well as sustainability-related factors such as CO_2_ emissions. Elsewhere, Kovács et al.^87^ developed methods to relate model predictions to both their current inputs and previously seen training data, using this to motivate new template- and scaffold-based splits. Focusing on the inverse direction (i.e., retrosynthesis) instead, Yu et al.^90^ developed OOD template- and size/scaffold-based splits to evaluate models on different forms of extrapolation; we discussed how the splits we consider can be viewed in a similar framework in the previous section. While these works share much of our philosophy—of trying to better characterize current reaction predictors’ already existing generalization abilities—they do not consider the breadth of different OOD tasks we consider here. Therefore, working out how to incorporate our splits into new benchmarks, such as that proposed by Gil et al.,^56^ would make an interesting future direction.

### Interplay between Model and Data

We have argued that a key factor in designing better evaluations is to consider the provenance of the data and its structure. While here this has involved considering the human aspect of data set curation (Figure 2), further down the line an optimal prospective study might introduce how an ML-based model additionally interacts with the data-generating process.^91^ In considering such a benchmark there will always be trade-offs to be made between the faithfulness of the setup versus the practicalities of being able to run it.

### Splits Are Useful in Unison

Ultimately, there is not a single “best” type of split to use when evaluating reaction predictors. In the previous section, we analyzed how the splits differed and discussed how they can be linked to particular modeling objectives. However, even within a single objective it is important to consider multiple splits. For instance, when evaluating if a model can correctly “discover” a new reaction, neither time-based splits or reaction-type splits capture the full picture alone: time-based splits also represent other shifts such as substrate changes, while reaction-type splits ignore aspects of the real causal discovery process. (For instance, imagine that the reaction discovery process has proceeded in the reaction type order *A* → *B* → *C*, and that we are holding out and evaluating on reaction type *B*. Then the model can use both the information from reaction types *A* and *C* when making this prediction, even though the latter information was not available during reaction type *B*’s real discovery.) Therefore, different splits offer complementary information about model capabilities and it is useful to use them together.

## Conclusion

In this work, we have highlighted the overoptimistic nature of current reaction predictor evaluations due to their unrealistic in-distribution setting, and discussed and evaluated ways to build better benchmarks. We have shown how document-based splits better account for the inherent factored structure of existing data sets, time-based splits enable prospective evaluation of a model’s performance, and reaction-type splits can assess a model’s capability for reaction discovery. By providing more faithful measures of current reaction predictor performance for different use cases, we draw attention to areas where they can be improved, to enable these shortcomings to be addressed by next-generation models.

The benchmarks we propose can also be further improved and developed. For instance, we only consider patent data here, which may have some key differences to data sets derived from academic papers or high throughput experimentation. Other future directions to pursue include exploring additional reaction-type splits, investigating how models might adapt quickly (e.g., using in-context learning), as well as analyzing how modeling choices might affect generalization. Exploring these modeling choices could include looking both at the architectural details of the model (e.g., SMILES versus graph representation schemes) and higher-level aspects such as the benefits of encoding reaction mechanistic information in the learning task.

Looking forward, we view reaction discovery as one of the most appealing use cases for reaction predictors. Although additional techniques are required to build a practical system (e.g., a method of sampling which molecules to feed through to a predictor), our more representative evaluation settings—and the insights they deliver—act as an important component of progressing toward this goal.

## Data Availability

The Pistachio data set (version 2022Q4) used to derive the splits in this study was obtained from NextMove Software (https://www.nextmovesoftware.com/); our code to reproduce the splits (assuming one has access to the Pistachio data set) is available at https://github.com/john-bradshaw/rxn-splits.

## Supporting Information Available

Additional details about reaction data set construction, splits, and model architecture; additional experimental results (PDF)
