# Supplementary material for: Challenging Reaction Prediction Models to Generalize to Novel Chemistry
Source: ACS Cent Sci. 2025 Mar 12;11(4):539–49. doi: 10.1021/acscentsci.5c00055 (PMC12022916; doi:10.1021/acscentsci.5c00055)
Supplement: Supplementary file 1 — oc5c00055_si_001.pdf [file oc5c00055_si_001.pdf]

Supplementary Material for "*Challenging reaction prediction models to generalize to novel chemistry*"

John Bradshaw,[†,‖] Anji Zhang,[†] Babak Mahjour,[†] David E. Graff,[†,‡,‖] Marwin H.S. Segler,[¶] and Connor W. Coley[*,†,§]

†*Department of Chemical Engineering, Massachusetts Institute of Technology, Cambridge, MA 02139, United States*

‡*Department of Chemistry and Chemical Biology, Harvard University, Cambridge, MA 02138, United States*

¶*Microsoft Research AI for Science, Cambridge CB1 2FB, United Kingdom*

§*Department of Electrical Engineering and Computer Science, Massachusetts Institute of Technology, Cambridge, MA 02139, United States*

‖*Present Address: Prescient Design, Genentech, South San Francisco, CA 94080, United States*

E-mail: ccoley@mit.edu

S1 Methods

In this section we describe further details about the data and models we use. Specifically, we first describe the dataset used and how this is pre-processed, and then we go on to describe the individual splits. Section S1.5 describes how we tune, train, and evaluate our models, and Section S1.6 provides further details of how plots in the main text were created. Further methodological details can be found in our code, available online at:

`https://github.com/john-bradshaw/rxn-splits`: for cleaning the datatset and creating our splits (i.e., the processes described in §S1.1–S1.4).

`https://github.com/john-bradshaw/rxn-lm`: for training and evaluating our models (§S1.5).

S1.1 Creating a clean reaction dataset

Our dataset and splits are created from the 2022Q4 version of the Pistachio dataset[1,2] (although not presented here, we also performed similar experiments with earlier versions of this dataset, finding similar qualitative trends). We restrict ourselves to the *US grants* part of this dataset (as opposed to for instance the *applications* part) to limit the same reactions coming up from different jurisdictions and filings. To create a cleaned dataset from these reactions, we perform three steps: (1) standardization, (2) filtering, and (3) deduplication, the details of which we go into below. While these steps likely do not remove all incorrectly reported reactions, they weed out a number of strange reactions and ensure that reactions that are completely identical cannot occur in both the training and test sets. We leave investigations into extending these steps and better dealing with missing ground truth details (e.g., additional products created) as future directions to explore.

(1) Standardization. To standardize reactions we remove any atoms maps and canonicalize the molecules using RDKit[3] (if the canonicalization fails then we skip the reaction). We also remove any ChemAxon SMILES extensions,[a] but keep any stereochemical information that is encoded directly in the SMILES (e.g., using the symbols '`\`', '`@`', etc). Reagents (i.e., molecules present but not contributing heavy atoms to our products) are mixed with reactants, and in general we make no distinction between these and ordinary reactants in our experiments.

(2) Filtering. Going through our standardized reactions, we then filter out any reactions that do not meet a certain set of criteria. The aim of these criteria are to identify both strange

[a] See `https://docs.chemaxon.com/display/docs/formats_chemaxon-extended-smiles-and-smarts-cxsmiles-and-cxsmarts.md`

reactions (e.g., those involving very large molecules) and non-interesting reactions (e.g., those that only describe the disappearance of a reactant), which complicate any downstream analysis. Specifically, we remove any reactions for which any of the following conditions are true:

1. the reactants have fewer than 5 heavy atoms,
2. the reactants contain no carbon atoms,
3. none of the reactants have at least two bonds,
4. the reaction is easily identifiable as a (de)protonation (we neutralize commonly occurring charged atoms in the reactants and products using a SMARTS pattern and, having done so, see if the sets of reactants and products are then equal),
5. all products not already present in the reactant set contain fewer than 2 heavy atoms,
6. the reaction is very long when tokenized[b] for our language model–based reaction predictor (e.g., over 800 tokens long).

(3) Deduplication. Finally, we deduplicate reactions by putting each reaction's reactant(s)-product(s) pair into a canonical representation. When picking between duplicates, we typically keep the first one we encounter when iterating through the Pistachio dataset; however, we displace the first one if it is missing a NameRxn tag (and the subsequent reaction we encounter is not) or if, failing this, the year associated with the subsequent reaction is earlier than the year associated with the first reaction we encountered. This is so that we try to keep the earliest recorded complete occurrence of each reaction. While this deduplication routine is not perfect, it ensures that at test time we do not evaluate on an example that completely matches one in the training set.

[b]Details on the tokenizer we use can be found in §S1.5.

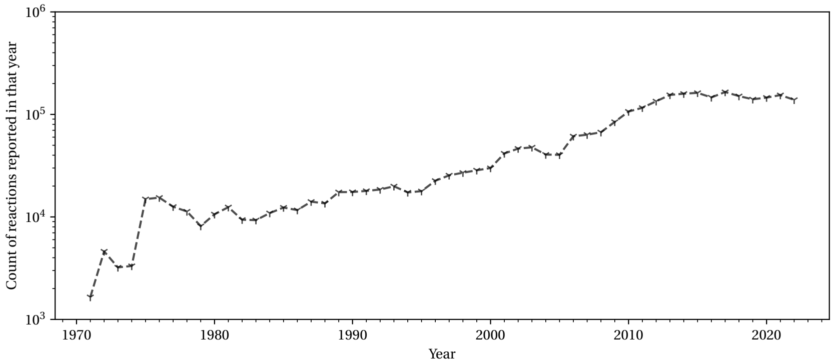

Figure S1: The number of reactions associated with each year in our cleaned data from 1971–2022. We refer interested readers to the works of Schneider et al.[4], Roughley and Jordan[5] for further details on how the distribution of reactions recorded in typical datasets has evolved over time.

In total we end up with just over 2.8mn (million) unique reactions. These are used as a starting point for creating our individual splits. Overall, these reactions come from over 200k (thousand) different documents (where each document is distinguished by the first part of the patent number), representing the work of just under 180k different authors spread across nearly 11k different assignees. The number of reactions associated with each year in our final deduplicated dataset is presented in Fig. S1. One can see that generally the number of reactions reported per year increases over time.

S1.2 Document- and author-based splits

In our document- and author-based splits we create an ID training set size of 1mn reactions, and three test sets of 100k reactions each: an ID and two OOD test sets (we also create an ID validation set for hyperparameter tuning containing 30k reactions). This is done in a series of steps, the key parts of which are detailed below. Note that we use the document title (as it exists in Pistachio) when referring to documents, but our process could also be extended in the future to tie together related documents, for instance by using patent citation information.

(1) Defining author-to-document and document-to-reaction maps. First, we take our cleaned, processed dataset (described in the previous section) and define a mapping from authors to

documents (a many-to-many relationship) and a mapping from documents to reactions (a one-to-many relationship due to the deduplication process described in Section S1.1).

(2) Splitting on authors. Using our constructed mappings, we then create our author-based split. To do this we iterate through our list of possible authors (in a random order) and assign all the associated documents (if they have not been encountered already) first to an OOD author set until that is full (i.e., contains more than 100k reactions to create the author-based test set) and then to an ID author set until that is full (i.e., contains more than 1230k reactions to create our remaining training and test sets—described next).

(3) Splitting on documents. The documents associated with the ID author set are further divided into two. This happens in a similar manner to how the authors were separated: we iterate through our list of documents (in a random order) and assign all the associated reactions first to an OOD document set until that is full (i.e., contains more than 100k reactions to create the document-based test set) and then to an ID document set until that is full (i.e., contains more than 1130k reactions to create our ID sets). The reactions in the ID document set are randomly divided into the ID sets: 1mn for the training set, 100k for the ID test set, and 30k for an ID validation set (used for hyperparameter tuning).

The end result of this, as we mentioned above, is three separate test sets, each 100k reactions in size (one split *on authors*, one *on documents*, and one only *on reactions*), as well as a 1mn reactions training set and a 30k reactions validation set. In total, we use 1.33mn reactions from our cleaned dataset (47% of the total) to form all the training/validation/test sets used in the author- and document-based splits; these reactions represent the work of just over 100k authors (56% of the total) and just over 72k documents (36% of the total).

Note that our splitting procedure means that the datasets are subtly different from sub-sampled reaction sets considered elsewhere: they are "author-document-dense." By "author-document-dense," we mean that when forming the sets, we try to use the minimum possible

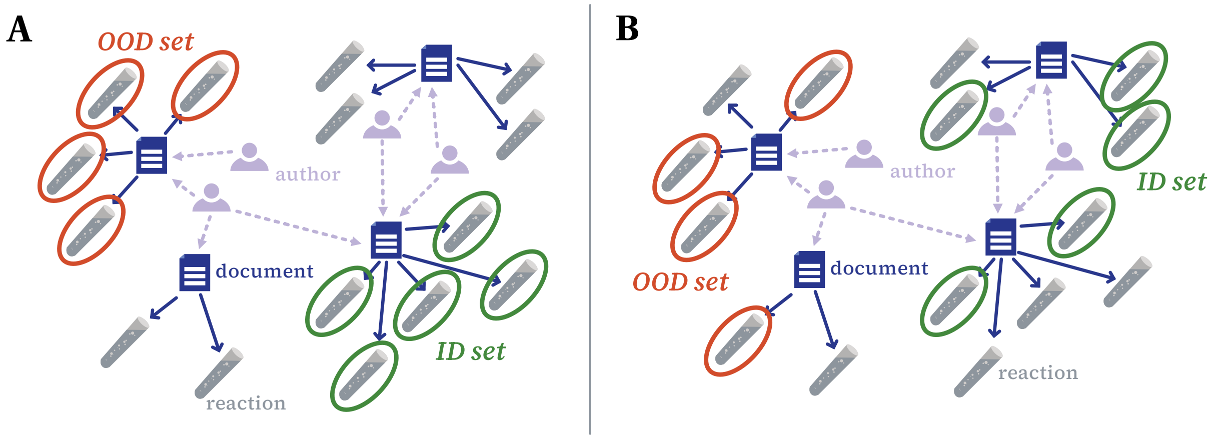

Figure S2: The splitting procedure we use is more likely to form a document-dense split (panel A), where the OOD set (shown in red) and ID set (shown in green) are sampled from a small number of documents—see text for further details. While other document-based splits could also be considered (such as a split similar to that shown in panel B), our splitting procedure has the advantages of being data efficient and also reflecting the author-document-reaction structure found in the complete dataset (i.e., without any subsampling).

number of authors and documents necessary. In other words, if one reaction from a document ends up in our OOD document test set, then all the other reactions associated with that document are *very likely*[c] to as well (Fig. S2). Ultimately, this may mean that our sets represent a smaller total area of chemical space than from a random subsample.[d] This observation highlights a central argument of our work: when considering the performance of reaction predictors, it is not enough to simply consider the size of the datasets they are trained on—it is critical to bear in mind their provenance too.

S1.3 Time-based splits

The time-based splits are created from our cleaned dataset by first ordering all reactions by year. Starting at 1976, we break off a held-out test set of 3k reactions for each year. (When creating these splits we use a document-based splitting strategy—we explain why below.) The remaining

[c]We use the term *very likely* as some reactions will be discarded from the list of OOD reactions to get back down to a 100k test set when finalizing the datasets.

[d]In a random subsample one could model the counts of reactions taken from each document as following a multivariate hypergeometric distribution.

reactions are used to create a separate training and validation set for each cutoff; each of these contains reactions that were reported up to (including in) the designated year. We create the training/validation sets for each cutoff independently (i.e., the reactions in the training set for an earlier cutoff do not influence the reactions chosen for the training set in a later cutoff).

When producing the time-based split results reported in the main paper, we control for dataset size. The validation sets each contain 2k reactions, and the training set sizes are just under 250k reactions each—this is the maximum available reactions we could use in the earliest split. In Section S2.2, we examine time-based splits that do not control for training set size (the validation set still comprises 2k reactions); here, the training set size is still just under 250k reactions for the 1996 cutoff set (this represents all that are available), but rises to approximately 2.26mn reactions for the final 2020 cutoff set.

Using a document-based splitting strategy when creating test sets. As mentioned above, when creating the test sets for each year, we use a document-based splitting strategy. This is because a time-based split implicitly creates document-type splits after the model's cutoff point (due to each document being associated with only one publication year). Without a document-based spitting strategy elsewhere, this transition manifests as a sharp drop in accuracy at the cutoff point. This drop complicates our primary analysis into investigating the extrapolation difficulty due to distribution shifts over time. Therefore, to maintain consistency, we employ a document-based splitting strategy when creating the held-out test set for every year.

Creating the Buchwald–Hartwig test set. To create the Buchwald-Hartwig test set (Fig. 3B), we go back through our cleaned dataset and extract all of the Buchwald-Hartwig reactions that were not included in our previously created training and validation sets. This process results in approximately 16k Buchwald-Hartwig reactions in total, corresponding to the NameRxn codes "*1.3.1*", "*1.3.2*", "*1.3.3*", "*1.3.4*", and "*1.9.43*".

S1.4 Reaction-type splits

As outlined in the main text, we created the reaction-type splits using the NameRxn classification system. The NameRxn code for each reaction is provided as part of the Pistachio dataset. To create our splits using these, we first removed all uncategorized reactions (with NameRxn code "*0.0*") from our cleaned dataset, to avoid inadvertently training on reactions that might be similar to those that we are trying to exclude (note that this means that the accuracy results for our reaction-type splits are not directly comparable to the other splits considered). The remaining reactions are then split independently six times: five times with our different held-out reaction types and once holding out no reaction types at all (this "base" split is used for hyperparameter tuning). The following NameRxn classes are used for each split:

Grignard Ester: "*3.7.14 Bromo Grignard + ester reaction*", "*3.7.15 Chloro Grignard + ester reaction*", "*3.7.17 Iodo Grignard + ester reaction*", and "*3.7.19 Grignard ester substitution*".

Heck: "*3.2 Heck reaction*" (including all subclasses).

Chloro Suzuki: "*3.1.2 Chloro Suzuki coupling*" and "*3.1.6 Chloro Suzuki-type coupling*".

Triflyloxy Suzuki: "*3.1.4 Triflyloxy Suzuki coupling*" and "*3.1.8 Triflyloxy Suzuki-type coupling*".

All Suzuki: "*3.1 Suzuki coupling*" (including all subclasses).

Each split creates five different sets: a 1mn reactions training set, a 10k reactions validation set, a 10k reactions ID test set, and two OOD reaction sets made up of only reactions from the held-out reaction classes. The first OOD set contains 1000 reactions and is added to the training set when training the baseline model for that split (see main text for details); the second, containing what remaining OOD reactions are available (and capped at a maximum 10k in size), is used as our OOD test set. As a result mainly of the amounts of different reactions available in our cleaned dataset, the OOD test set is approximately 1k reactions in size for the Grignard Ester split, 2k reactions in size for the Heck split, 3k in size for the Triflyloxy Suzuki split, and 10k in size for the other splits. (We emphasize that only the test set size differs for the different splits,

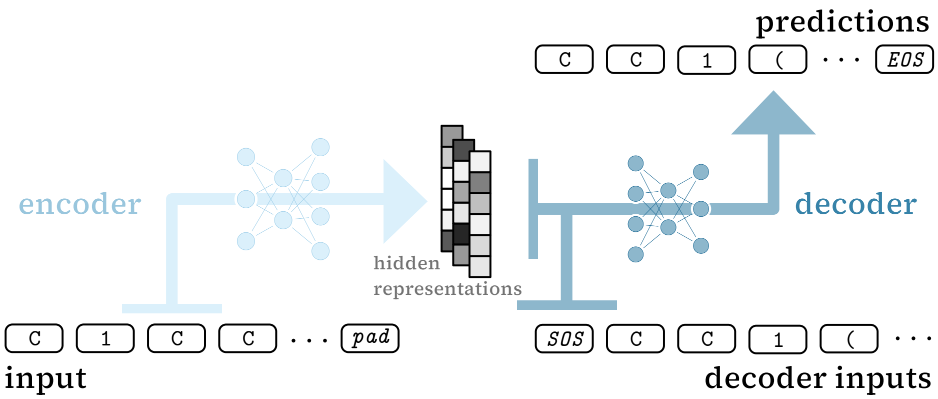

Figure S3: We use an encoder-decoder Transformer model for reaction prediction based on the BART architecture.[6] SMILES are tokenized using the scheme proposed by Schwaller et al.[7] and special tokens (e.g., for *padding*, *start of sequence*, and *end of sequence* are added as appropriate). Both the encoder and decoder use attention mechanisms to focus on different parts of the initial input (i.e., the reactants) and decoder inputs (i.e., the parts of the products predicted so far).

the training set size is kept constant.) When creating the different reaction-type splits, we use a document-based splitting strategy for consistency with the earlier experiments.

The NameRxn system is helpful for our use case due to its good coverage (74% of our cleaned dataset has been categorized) and hierarchical nature (meaning we can split at different levels). However, it is not the only classification system suitable for assessing the ability of reaction predictors to generalize to new reaction types. Different classification systems will likely have different classification rules and different relationships between the classes they consider, leading to other advantages and disadvantages, and so could be an interesting future direction to explore.

Identifying single and double additions in the Grignard Ester split. For the analysis in the section entitled "Deeper investigation into what enables reaction class extrapolation", we further split the Grignard Ester set down into single and double addition subsets. This was done by writing out a SMARTS pattern to count the number of alcohol groups present in the products versus the reactants. While such an approach may produce a few false positives, we found this practical method simple and generally effective.

S1.5 Model

For the experiments, we use an encoder-decoder Transformer model[8] based on the BART architecture[6] (Fig. S3). Specifically, we use the implementation from HuggingFace's Transformer library,[9] but with the SMILES tokenization scheme[e] proposed by Schwaller et al., §3.1,[7] as opposed to byte-pair encoding. (Generally we expect the difference in tokenization to have a minor effect; see Chithrananda et al., §4.1,[10] for a discussion on the differences in molecular tokenization performance for a molecule representation task). We do not pretrain our model on a denoising task, instead training from scratch for each task in a supervised manner only. Overall the model used is very similar to Schwaller et al.'s Molecular Transformer.[7]

Training and evaluation

In order to ensure that the hyperparameters used for the model are suited to each task, we run hyperparameter optimization using Ray Tune.[12] For the document- and author-based splits, the tuning is run on the *on reactions* split; for the time-based split, it is run on the 1996 cutoff split; and for the reaction-type splits, it is run on the *base* split (see §S1.4). The hyperparameters we tune, along with their given ranges, are shown in Table S1; when tuning, we use an ASHAScheduler,[13] optimize for the loss on the validation set, and consider 100 potential different trials. We stick to using models that are able to fit on a single GPU (we predominantly use NVIDIA RTX A5000 and NVIDIA GeForce RTX 3090 GPUs with approximately 24GB of memory). Therefore, any hyperparameter combinations that cause the model to run out of GPU memory during training are discarded at the end.

In general, we use early stopping when training our final models for each experiment using our optimized hyperparameters. Early stopping is done using the loss on our validation set; this does not always perfectly correlate with accuracy, but it is fast to compute due to the fact that it can be done on each token in parallel using teacher forcing. When evaluating our models, we use

[e] We modify this scheme slightly to extend it to cover the large loop numbers that can occur in the SMILES of the Pistachio dataset.

Table S1: Grid used for hyperparameter tuning on the Pistachio dataset splits. Please see our code (`https://github.com/john-bradshaw/rxn-lm`) for further details.

**Hyperparameter**	**Grid**	**Note**
Gradient accumulation steps	$\{2,4,8,16\}$	We use a fixed batch size and control the effective batch size using gradient accumulation (so that larger batches can fit in GPU memory).
Learning rate	$\mathsf{loguniform}(1e-5, 1e-2)$	We use the AdamW optimizer.[11]
Warmup steps	$[100, 10000]$	We use a cosine scheduler with linear warmup.
Encoder layers	$\{2,3,4,\ldots,12\}$	
Encoder FFN dim.	$\{512,1024,2048,4096\}$	The dimension of the intermediate layer for the feedforward network in the encoder.
Encoder attention heads	$\{4,8,16,32\}$	Number of attention heads in the encoder.
Decoder layers	$\{2,3,4,\ldots,12\}$	
Decoder FFN dim.	$\{512,1024,2048,4096\}$	The dimension of the intermediate layer for the feedforward network in the decoder.
Decoder attention heads	$\{4,8,16,32\}$	Number of attention heads in the decoder.
Dim. model	$\{128,256,512,1024\}$	Excludes the layers listed above for which the dimension is set separately.
Dropout	$[0.0, 0.6]$	The dropout probability in the embeddings, pooler, and encoder for the fully connected layers.

beam search (with a width of 5) and compare the predicted SMILES to the ground truth SMILES after canonicalization.

We wish to stress that it is likely that the models we train do not obtain state-of-the-art accuracy on the tasks we consider. Various avenues can be explored to improve performance further, such as considering larger multi-GPU models, performing more extensive hyperparameter tuning, training for different amounts of time, and using SMILES augmentation or Polyak averaging (Ref. 14, §8.7.3) (both of which have previously been shown to help similar models[15]).[f]

[f]Having said that, we ensured that our method was able to obtain comparable results with the Molecular Transformer on the augmented USPTO-MIT dataset (Ref 15, Table 3); here, we found our model obtained a top-1

However, obtaining the absolute best performing model is not the aim of our work, and during experiments with slightly different models, datasets, and even training regimes, we found that the qualitative trends we observed remained fairly consistent even if exact accuracy values differed.

S1.6 Creating Fig. 6

Fig. 6 in the main text was created by calculating (for each test reaction) the average cosine distance (in reactant and reaction fingerprint space) to the nearest 5 neighbor reactions in the corresponding split's training set. To create this plot we used radius 2 Morgan fingerprints with 2048 bits. Reagents (i.e., molecules that did not contribute atoms to the product) were removed from the reaction when computing fingerprints (otherwise the fingerprints tended to represent the common solvents, catalysts, etc used rather than the more unique characteristics of the reactions). The reaction fingerprints were calculated by computing the product fingerprints and then subtracting from these the reactant fingerprints:

$$\text{Reaction Fingerprint} = \texttt{fingerprint}(\text{products}) - \texttt{fingerprint}(\text{reactants}).$$

Note that the different groups of splits in this plot have slightly different setups, for instance, the time splits use far smaller training set sizes than the NameRxn splits. Aside from the *on reactions* split, all other splits use distinct documents for the train/test set. “NameRxn ID” is calculated with the in-distribution (ID) test set for the Grignard Ester split, but similar results are seen when using the ID test sets for the other NameRxn splits. Again note that this split is slightly different to the *on documents* split as reactions with NameRxn class “*0.0*” (i.e., uncategorized) are removed (see §S1.4 for further details).

accuracy of 87.1% and a top-5 accuracy of 94.3% when trained for 500k iterations.

S2 Further experimental results

This appendix contains further experimental results, complimenting those presented in the main text. Section S2.1 provides tables listing the main numerical results, while Section S2.2 provides further plots for the time-based splits.

S2.1 Tables

The tables provided here extend the main accuracy results presented in the main text. In particular, Table S2 provides top-1 through top-5 accuracies for the document- and author-based splits (see Fig. 2), Table S3 provides the same for the Buchwald–Hartwig test set (see Fig. 3B), and Table S4 contains the accuracies for the NameRxn splits (see Fig. 4).

Table S2: Accuracies on the document- and author-based splits shown in Fig. 2.

**Split**	**top-1**	**top-2**	**top-3**	**top-4**	**top-5**
on reactions	0.65	0.72	0.74	0.76	0.77
on documents	0.58	0.65	0.68	0.69	0.70
on authors	0.55	0.62	0.64	0.66	0.67

Table S3: Accuracies on the Buchwald–Hartwig test set for the models trained on different time splits (see Fig. 3B). Note that the training set sizes for the time-based split models are smaller than those used for the other splits (as we control for the training set size across the different time cutoffs).

Split year	top-1	top-2	top-3	top-4	top-5	Num. of BH reactions in split's training set
1996	0.05	0.06	0.07	0.08	0.08	1
1998	0.08	0.11	0.13	0.14	0.14	5
2000	0.15	0.19	0.20	0.21	0.22	26
2002	0.15	0.19	0.20	0.21	0.22	53
2004	0.24	0.29	0.30	0.32	0.32	126
2006	0.28	0.32	0.34	0.35	0.36	195
2008	0.32	0.37	0.39	0.41	0.41	306
2010	0.39	0.44	0.46	0.48	0.48	520
2012	0.40	0.45	0.47	0.48	0.49	714
2014	0.49	0.55	0.57	0.58	0.59	1071
2016	0.55	0.60	0.62	0.63	0.64	1251
2018	0.64	0.70	0.72	0.73	0.74	1684
2020	0.62	0.68	0.69	0.70	0.71	1750

Table S4: Accuracies on the reaction type splits shown in Fig. 4. Note that the rows marked "w/1k OOD" are for the baseline models, where 1000 OOD reactions are added to the original training set to give a sense of the intrinsic difficulty associated with that reaction class.

Split	top-1	top-2	top-3	top-4	top-5
Grignard Ester	0.10	0.15	0.20	0.25	0.28
Grignard Ester w/1k OOD	0.85	0.89	0.91	0.92	0.93
Chloro Suzuki	0.74	0.82	0.85	0.87	0.88
Chloro Suzuki w/1k OOD	0.81	0.88	0.90	0.91	0.92
Heck	0.07	0.19	0.29	0.36	0.40
Heck w/1k OOD	0.63	0.81	0.86	0.88	0.89
All Suzuki	0.51	0.62	0.67	0.70	0.72
All Suzuki w/1k OOD	0.76	0.83	0.86	0.87	0.89
Triflyloxy Suzuki	0.83	0.90	0.92	0.93	0.94
Triflyloxy Suzuki w/1k OOD	0.85	0.91	0.93	0.93	0.94

S2.2 Additional time-based split results

This section contains more results for the time-based splits. Fig. S4 is the complement of Fig. 3A in the main text, showing the equivalent top-5 accuracy (the figure in the main text shows the top-1 accuracy) for the models trained on different time splits, when we control for training set size.

Fig. S5 and Fig. S6 show the top-1 and top-5 accuracies respectively for an experiment where we no longer control for training set size when forming the splits. Specifically, for each time cutoff, we use all of the available reactions (after removing those used to form the held-out test sets) to train each model. This means that models trained on sets associated with later time cutoffs will have seen far more reactions than models trained on the earlier ones. While this approach may better reflect how these models are updated in the real world, it entangles the effects of different dataset sizes with changing data distributions.

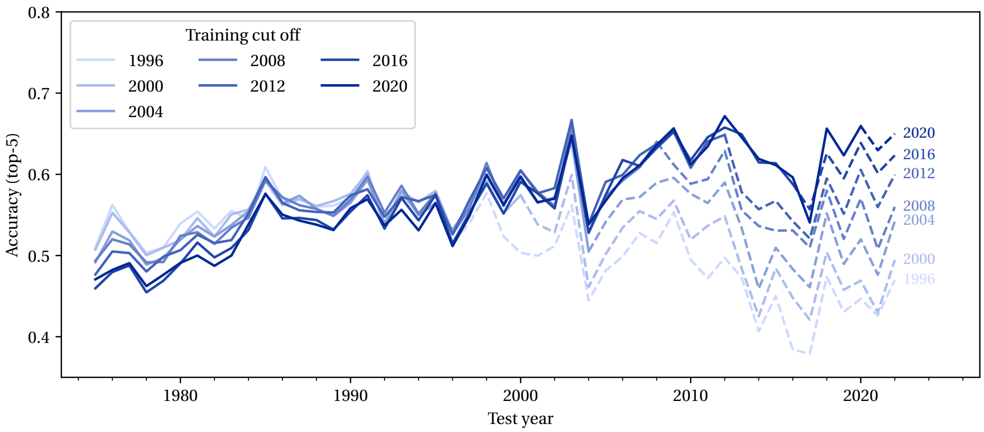

Figure S4: Top-5 accuracy for reaction predictors trained up to different timepoints (different colors) when evaluated on held-out test sets for each year (x-axis). For instance the line in the lightest shade, marked “1996”, reports the top-5 accuracy for a reaction predictor trained on reactions that were reported up to and including 1996. The dashed line indicates model performance when the model is “extrapolating”, in this context meaning that the test set year is beyond those associated with the reactions seen in the model’s training set. Similar to Fig. 3A in the main paper, we control for training set size (so each model sees the same number of reactions in training).

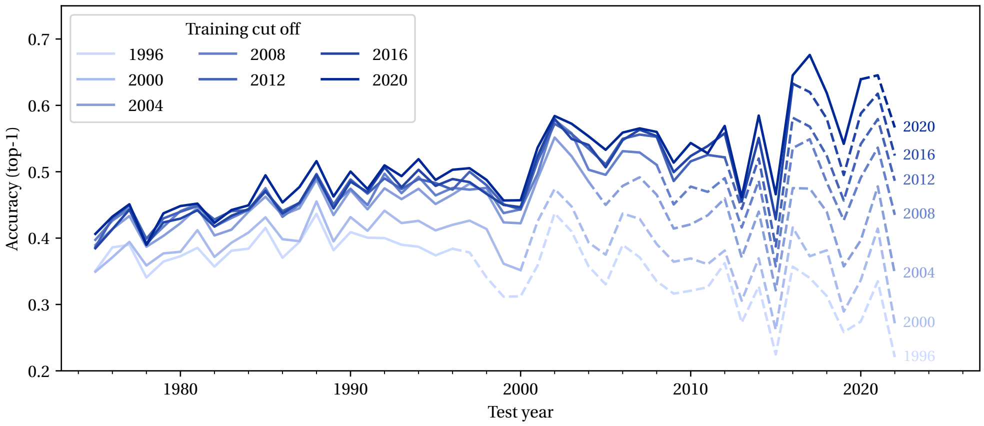

Figure S5: Top-1 accuracy for reaction predictors trained up to different timepoints (different colors) when evaluated on held-out test sets for each year (x-axis). For instance, the line in the lightest shade, marked "1996", reports the top-1 accuracy for a reaction predictor trained on reactions that were reported up to 1996 (inclusive). The dashed line indicates model performance when the model is "extrapolating"—meaning that the test set year is beyond those associated with the reactions seen in the model's training set. Note unlike Fig. 3A in the main paper, **we do not control for training set size** for these models (so each model sees a different number of reactions in training).

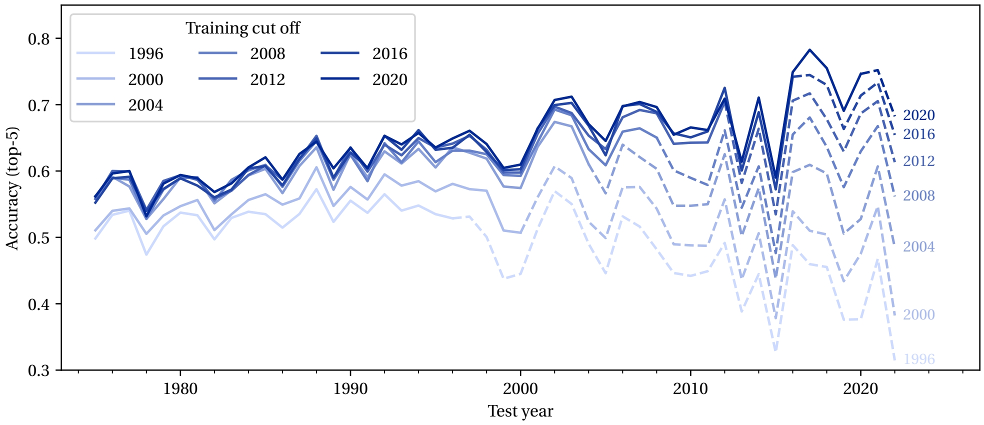

Figure S6: Top-5 accuracy for reaction predictors trained up to different timepoints (different colors) when evaluated on held-out test sets for each year (x-axis). For instance, the line in the lightest shade, marked "1996", reports the top-5 accuracy for a reaction predictor trained on reactions that were reported up to 1996 (inclusive). The dashed line indicates model performance when the model is "extrapolating"—meaning that the test set year is beyond those associated with the reactions seen in the model's training set. Note unlike Fig. 3A in the main paper, **we do not control for training set size** for these models (so each model sees a different number of reactions in training).

References

(1) Mayfield, J.; Lowe, D.; Sayle, R. Pistachio - Search and Faceting of Large Reaction Databases. ACS Fall 2017, 2017; https://nextmovesoftware.com/talks/Mayfield_Pistachio_NIHReactions_202105.pdf.

(2) NextMove Software Pistachio. https://www.nextmovesoftware.com/pistachio.html, 2021; Accessed: 2021-11-18.

(3) RDKit Team RDKit: Open-source cheminformatics. 2021; http://www.rdkit.org.

(4) Schneider, N.; Lowe, D. M.; Sayle, R. A.; Tarselli, M. A.; Landrum, G. A. Big Data from Pharmaceutical Patents: A Computational Analysis of Medicinal Chemists' Bread and Butter. *Journal of medicinal chemistry* **2016**, *59*, 4385–4402, DOI: doi:10.1021/acs.jmedchem.6b00153.

(5) Roughley, S. D.; Jordan, A. M. The medicinal chemist's toolbox: An analysis of reactions used in the pursuit of drug candidates. *Journal of medicinal chemistry* **2011**, *54*, 3451–3479, DOI: doi:10.1021/jm200187y.

(6) Lewis, M.; Liu, Y.; Goyal, N.; Ghazvininejad, M.; Mohamed, A.; Levy, O.; Stoyanov, V.; Zettlemoyer, L. BART: Denoising Sequence-to-Sequence Pre-training for Natural Language Generation, Translation, and Comprehension. Proceedings of the 58th Annual Meeting of the Association for Computational Linguistics. 2020; pp 7871–7880, DOI: doi:10.18653/v1/2020.acl-main.703.

(7) Schwaller, P.; Gaudin, T.; Lanyi, D.; Bekas, C.; Laino, T. "Found in Translation": Predicting Outcomes of Complex Organic Chemistry Reactions using Neural Sequence-to-Sequence Models. *Chemical science* **2018**, *9*, 6091–6098, DOI: doi:10.1039/C8SC02339E.

(8) Vaswani, A.; Shazeer, N.; Parmar, N.; Uszkoreit, J.; Jones, L.; Gomez, A. N.; Kaiser, L.; Polosukhin, I. Attention Is All You Need. Advances in Neural Information Processing Systems 30. 2017; pp 5998–6008.

(9) Wolf, T. et al. HuggingFace's Transformers: State-of-the-art Natural Language Processing. *arXiv [cs.CL]* **2019**, DOI: doi:10.48550/arXiv.1910.03771.

(10) Chithrananda, S.; Grand, G.; Ramsundar, B. ChemBERTa: Large-Scale Self-Supervised Pretraining for Molecular Property Prediction. Machine Learning for Molecules Workshop at NeurIPS 2020. 2020; DOI: doi:10.48550/arXiv.2010.09885.

(11) Loshchilov, I.; Hutter, F. Decoupled weight decay regularization. International Conference on Learning Representations 2019. 2019.

(12) Moritz, P.; Nishihara, R.; Wang, S.; Tumanov, A.; Liaw, R.; Liang, E.; Elibol, M.; Yang, Z.; Paul, W.; Jordan, M. I.; Stoica, I. Ray: A distributed framework for emerging AI applications. 13th USENIX Symposium on Operating Systems Design and Implementation (OSDI 18). 2018; pp 561–577.

(13) Li, L.; Jamieson, K.; Rostamizadeh, A.; Gonina, E.; Ben-tzur, J.; Hardt, M.; Recht, B.; Talwalkar, A. A System for Massively Parallel Hyperparameter Tuning. Proceedings of Machine Learning and Systems. 2020; pp 230–246.

(14) Goodfellow, I.; Bengio, Y.; Courville, A. *Deep Learning*; The MIT Press, 2016.

(15) Schwaller, P.; Laino, T.; Gaudin, T.; Bolgar, P.; Hunter, C. A.; Bekas, C.; Lee, A. A. Molecular Transformer: A Model for Uncertainty-Calibrated Chemical Reaction Prediction. *ACS Central Science* **2019**, *5*, 1572–1583, DOI: doi:10.1021/acscentsci.9b00576.
